# Trends in Diagnosed Attention-Deficit/Hyperactivity Disorder Among Children, Adolescents, and Adults in Japan From April 2010 to March 2020

**DOI:** 10.1001/jamanetworkopen.2022.34179

**Published:** 2022-09-30

**Authors:** Daimei Sasayama, Rie Kuge, Yuki Toibana, Hideo Honda

**Affiliations:** 1 Department of Psychiatry, Shinshu University School of Medicine, Matsumoto, Nagano, Japan; 2 Department of Child and Adolescent Developmental Psychiatry, Shinshu University School of Medicine, Matsumoto, Nagano, Japan; 3 Mental Health Clinic for Children, Shinshu University Hospital, Matsumoto, Nagano, Japan

## Abstract

This cohort study assesses trends in incidence of attention-deficit/hyperactivity disorder (ADHD) among children, adolescents, and adults in Japan from 2010 to 2019.

## Introduction

Attention-deficit/hyperactivity disorder (ADHD) is one of the most common neurobehavioral disorders of childhood.^1^ Approximately 50% of childhood cases persist into adulthood.^2^ However, adult ADHD is often underdiagnosed owing to inadequate recognition of the disorder.^3^ We used the National Database of Health Insurance Claims and Specific Health Checkups of Japan (NDB) to examine trends in the nationwide incidence of newly diagnosed ADHD from April 2010 to March 2020.

## Methods

This cohort study was approved by the ethics committee of Shinshu University School of Medicine. Informed consent was not required because anonymous data were used. This study followed the STROBE reporting guideline. Demographic and diagnostic information was collected from the NDB, which includes all electronic health insurance claims from April 2009 to March 2020. The NDB is described on the Ministry of Health, Labour and Welfare website^4^ and in our previous study.^5^

Data on individuals newly diagnosed with ADHD (*ICD-10* codes F90.0, F90.1, and F98.8) between fiscal years (FY) 2010 and 2019 were retrieved. Extracted information were sex and the year and age group at diagnosis. Incidence in each age group was calculated by dividing the number of ADHD diagnoses by the total population in the age group. Microsoft Excel 2016 was used for analysis. Details are given in the eMethods in the Supplement.

## Results

A total of 838 265 individuals were newly diagnosed with ADHD between FY 2010 and 2019. Of 121 278 individuals diagnosed at age 0 to 6 years, 23 292 (19.2%) were female and 97 986 (80.8%) were male. Of 381 753 individuals diagnosed at age 7 to 19 years, 91 891 (24.1%) were female and 289 862 (76.0%) were male. Of 335 234 individuals diagnosed after age 19 years, 160 239 (47.8%) were female and 174 995 (52.2%) were male.

The Figure shows the incidence of newly diagnosed ADHD in each age group. Between FY 2010 and 2019, the incidence of newly diagnosed ADHD increased 2.7 fold among children aged 0 to 6 years (2.9 fold among females, 2.7 fold among males), 2.5 fold among those aged 7 to 19 years (3.7 fold among females, 2.2 fold among males), and 21.1 fold among adults older than 19 years (22.3 fold among females, 20.0 fold among males).

**Figure.**
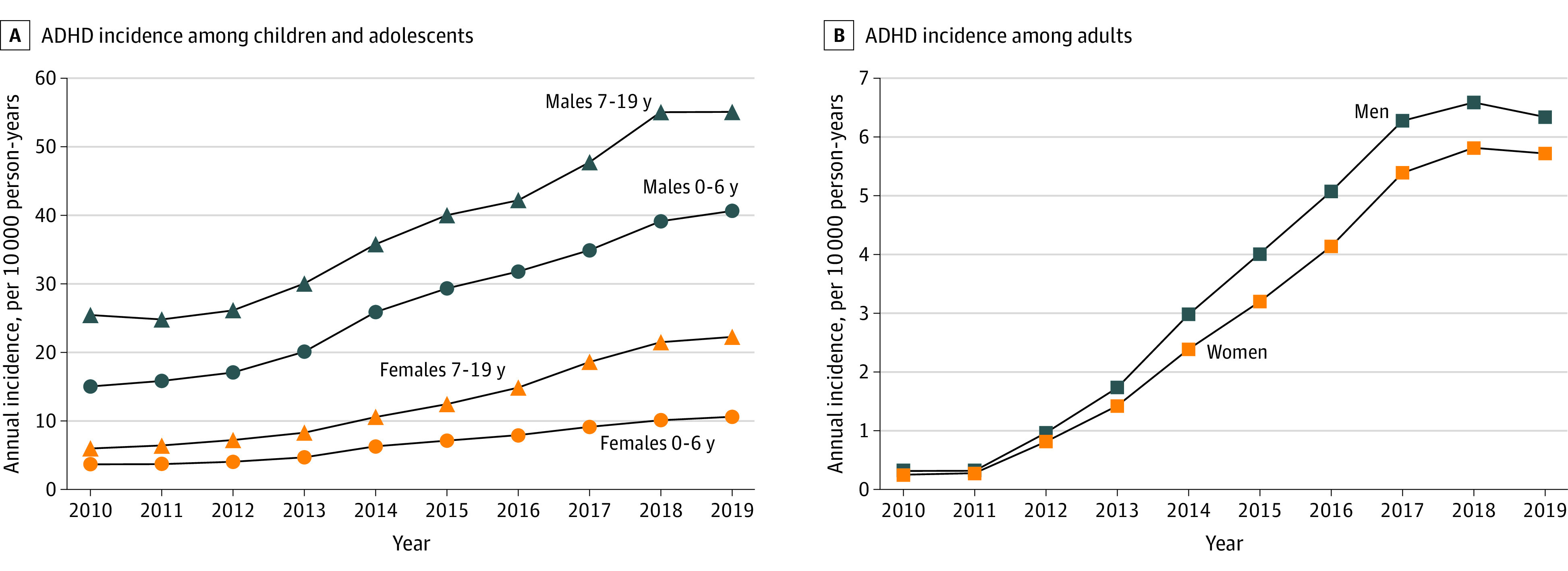
Annual Incidence of Attention-Deficit/Hyperactivity Disorder (ADHD) Among Children, Adolescents, and Adults in Japan Between 2010 and 2019

## Discussion

The incidence of newly diagnosed ADHD in Japan increased from 2010 to 2019. One reason may be the release of the *Diagnostic and Statistical Manual of Mental Disorders* (Fifth Edition) (*DSM-5*) in 2013, which allows combined diagnosis of ADHD and autism spectrum disorder (ASD). Although the *ICD-10* precludes a dual diagnosis of ADHD and ASD, the release of the *DSM-5* may have prompted physicians to give an additional diagnosis of ADHD to individuals with ASD to prescribe ADHD medications.

The increase was most prominent among adults between 2012 and 2017. This may be explained by increased sensitivity of diagnosis, especially of adult ADHD. The approval of the first medication in Japan in 2012 for adults with ADHD may have contributed to the expanding awareness of the disorder. The annual incidence of adult ADHD seems to have leveled after reaching a peak of 6 per 10 000 person-years in 2018. This figure is comparable to the adult ADHD incidence reported for Asian individuals in the US in 2016 (6.88 per 10 000 person-years).^6^ Thus, the sensitivity to detect ADHD in the adult population seems to have reached a standard in Japan.

Findings of this study are limited in that the available demographic data were restricted to sex and age group. Nevertheless, nonetiological factors could explain at least part of the apparent increase in ADHD incidence. Further investigation is warranted to elucidate how etiological and nonetiological factors contribute to the changing incidence of ADHD in Japan.

## Notes

Supplement.
eMethods.
