# Supplementary material for: Trends in Diagnosed Attention-Deficit/Hyperactivity Disorder Among Children, Adolescents, and Adults in Japan From April 2010 to March 2020
Source: JAMA Netw Open. 2022 Sep 30;5(9):e2234179. doi: 10.1001/jamanetworkopen.2022.34179 (PMC9526082; doi:10.1001/jamanetworkopen.2022.34179)
Supplement: Supplement. — eMethods. [file jamanetwopen-e2234179-s001.pdf]

Supplemental Online Content

Sasayama D, Kuge R, Toibana Y, Honda H. Trends in diagnosed attention-deficit/hyperactivity disorder among children, adolescents, and adults in Japan from April 2010 to March 2020. *JAMA Netw Open*. 2022;5(9):e2234179.
doi:10.1001/jamanetworkopen.2022.34179

eMethods.

This supplemental material has been provided by the authors to give readers additional information about their work.

© 2022 Sasayama D et al. *JAMA Network Open.*

eMethods

A retrospective cohort study was conducted using data from the national database of health insurance claims of Japan (NDB). The reporting of this study conforms to the Strengthening the Reporting of Observational Studies in Epidemiology (STROBE) statement for reporting of cohort studies. As described in our previous study,[1] the NDB includes all electronic-based health insurance claims in Japan since fiscal year 2009. The NDB includes the health insurance claims data such as sex, age or age group, and diagnostic codes. All permanent residents in Japan are mandatorily enrolled in the national universal health insurance program. Patients are free to choose any clinics or hospitals, and the health insurance covers 70-90% of their medical costs. The majority of medical care in Japan are covered by the national healthcare system and thus are included in the NDB. However, medical treatments not covered by public insurance and those completely covered by public funds are not included. A detailed description of NDB is described in the website of the Ministry of Health, Labour and Welfare.[2] Several retrospective, population-based studies have been conducted previously using the medical records retrieved from the NDB.[1,3,4]

Data on individuals diagnosed with ADHD (ICD-10 codes F90.0, F90.1, and F98.8 [attention deficit disorder without hyperactivity]) between fiscal years 2010-2019 were retrieved from the NDB. Extracted information were sex and the year and age group at diagnosis. To select only newly diagnosed cases, we extracted only the first recorded diagnosis of ADHD in the NDB (fiscal years 2009-2019) for each individual. Due to the absence of data before fiscal 2009, all recorded ADHD diagnoses in fiscal 2009 were counted as the first diagnosis in each individual. Therefore, many of the individuals recorded as first diagnosed in 2009 may have been previously diagnosed in 2008 or before. Thus, we did not include in our analyses the data on individuals diagnosed with ADHD in fiscal 2009.

Incidence for each year in each age group was calculated by dividing the number of

© 2022 Sasayama D et al. *JAMA Network Open.*

ADHD diagnosis by the total population of the age group. The national population data of October 1 in each fiscal year, retrieved from the data released by the Statistics Bureau of Japan,[5] were used for calculation.

The study was approved by the Ethics Committee of Shinshu University School of Medicine. Informed consent was not required due to the anonymous nature of the data.

References

1. Sasayama D, Kuge R, Toibana Y, Honda H. Trends in Autism Spectrum Disorder Diagnoses in Japan, 2009 to 2019. *JAMA Netw Open.* 2021;4(5):e219234.
2. Ministry of health labor and welfare Japan. On the use of the National Database of Health Insurance Claim Information and Specified Medical Checkups (in Japanese). 2013; http://www.mhlw.go.jp/stf/shingi/2r9852000002ss9z-att/2r9852000002ssfg.pdf.
3. Ono A, Ishikane M, Kusama Y, et al. The first national survey of antimicrobial use among dentists in Japan from 2015 to 2017 based on the national database of health insurance claims and specific health checkups of Japan. *PLoS One.* 2020;15(12):e0244521.
4. Okumura Y, Sakata N, Takahashi K, Nishi D, Tachimori H. Epidemiology of overdose episodes from the period prior to hospitalization for drug poisoning until discharge in Japan: An exploratory descriptive study using a nationwide claims database. *J Epidemiol.* 2017;27(8):373-380.
5. Statistics Bureau Ministry of Internal Affairs and Communications. Statistics Bureau of Japan. https://www.stat.go.jp/index.html. Accessed March 9, 2021.

© 2022 Sasayama D et al. *JAMA Network Open.*
